# A multi-center population-based case–control study of ovarian cancer in African-American women: the African American Cancer Epidemiology Study (AACES)

**DOI:** 10.1186/1471-2407-14-688

**Published:** 2014-09-22

**Authors:** Joellen M Schildkraut, Anthony J Alberg, Elisa V Bandera, Jill Barnholtz-Sloan, Melissa Bondy, Michelle L Cote, Ellen Funkhouser, Edward Peters, Ann G Schwartz, Paul Terry, Kristin Wallace, Lucy Akushevich, Frances Wang, Sydnee Crankshaw, Patricia G Moorman

**Affiliations:** Duke Cancer Institute, Department of Community and Family Medicine, Duke University Medical Center, Durham, NC USA; Hollings Cancer Center and Department of Public Health Sciences, Medical University of South Carolina, Charleston, SC USA; Cancer Prevention and Control Program, Rutgers Cancer Institute of New Jersey, New Brunswick, NJ USA; Case Comprehensive Cancer Center, Case Western Reserve University School of Medicine, Cleveland, OH USA; Cancer Prevention and Population Sciences Program, Baylor College of Medicine, Houston, TX USA; Department of Oncology, Karmanos Cancer Institute Population Studies and Disparities Research Program, Wayne State University School of Medicine, Detroit, MI USA; Division of Preventive Medicine, University of Alabama at Birmingham, Birmingham, AL USA; Epidemiology Program, Louisiana State University School of Public Health, New Orleans, LA USA; Departments of Public Health and Surgery, University of Tennessee-Knoxville, Knoxville, TN USA

**Keywords:** Epidemiology, Ovarian cancer, African American, Case–control study

## Abstract

**Background:**

Ovarian cancer (OVCA) is the leading cause of death from gynecological cancer, with poorer survival for African American (AA) women compared to whites. However, little is known about risk factors for OVCA in AA. To study the epidemiology of OVCA in this population, we started a collaborative effort in 10 sites in the US. Here we describe the study and highlight the challenges of conducting a study of a lethal disease in a minority population.

**Methods:**

The African American Cancer Epidemiology Study (AACES) is an ongoing, population-based case–control study of OVCA in AA in 10 geographic locations, aiming to recruit 850 women with invasive epithelial OVCA and 850 controls age- and geographically-matched to cases. Rapid case ascertainment and random-digit-dialing systems are in place to ascertain cases and controls, respectively. A telephone survey focuses on risk factors as well as factors of particular relevance for AAs. Food-frequency questionnaires, follow-up surveys, biospecimens and medical records are also obtained.

**Results:**

Current accrual of 403 AA OVCA cases and 639 controls exceeds that of any existing study to date. We observed a high proportion (15%) of deceased non-responders among the cases that in part is explained by advanced stage at diagnosis. A logistic regression model did not support that socio-economic status was a factor in advanced stage at diagnosis. Most risk factor associations were in the expected direction and magnitude. High BMI was associated with ovarian cancer risk, with multivariable adjusted ORs and 95% CIs of 1.50 (0.99-2.27) for obese and 1.27 (0.85- 1.91) for morbidly obese women compared to normal/underweight women.

**Conclusions:**

AACES targets a rare tumor in AAs and addresses issues most relevant to this population. The importance of the study is accentuated by the high proportion of OVCA cases ascertained as deceased. Our analyses indicated that obesity, highly prevalent in this population (>60% of the cases), was associated with increased OVCA risk. While these findings need to be replicated, they suggest the potential for an effective intervention on the risk in AAs. Upon completion of enrollment, AACES will be the largest epidemiologic study of OVCA in AA women.

## Background

Each year, over 22,000 new ovarian cancer (OVCA) cases are diagnosed in the United States, accounting for approximately 4% of cancers in women [1]. Epithelial OVCA is the most lethal gynecologic malignancy among both African American (AA) and white women, predominantly due to the absence of sufficiently accurate screening tests, resulting in most women having advanced disease at the time of clinical presentation [2]. Although incidence is lower among AA women than in white women (9.8 vs. 13.0 cases/100,000), 5-year relative survival is worse for AA women than white women across all ages (36% vs 44%) [3]. In addition, AA women tend to get the disease at a younger age (61 versus 64 years) [4].

Reasons for poorer survival among AA women are unknown [5], but are likely multi-factorial, including differences in treatment, access to care and comorbidities, as well as more aggressive presentation [6–9]. Preliminary data from our group and others suggest AA and white women with OVCA differ in the distribution of intrinsic subtypes associated with poorer outcome of ovarian cancer [10], in the prevalence of certain reproductive [11–13] and genetic risk factors [14–16], and in the receipt of guideline-recommended treatment [9].

Although currently available evidence is suggestive of differences in risk and prognostic factors between AA and white women, the evidence-base is limited. For example, the three epidemiologic studies reporting on risk factors for OVCA in AA women [11–13] all had fewer than 150 cases, reflecting the relatively small number of OVCA cases diagnosed in AA women and the barriers to enrolling a large number of cases from a single geographic location. With the goal of improving our understanding of factors that affect risk and survival among AA women with OVCA, we established the African American Cancer Epidemiology Study (AACES), an ongoing, multi-state, multi-center, population-based case–control study. The aims of this study include assessment of associations with established risk factors, evaluation of genetic susceptibility, characterization of tumor biology and evaluation of socioeconomic and behavioral factors that may affect prognosis through delays in diagnosis and treatment. The purpose of this paper is to describe the study design, challenges in recruitment, and the study population enrolled thus far.

## Methods

### Study design, subject identification and enrollment

The 10 AACES sites include institutions that are located in geographic regions with a relatively high density of AAs in the population and that have the capability of rapidly identifying newly diagnosed cases of OVCA. The geographic regions are largely concentrated in the southern US (Alabama, Georgia, Louisiana, North Carolina, South Carolina, and Tennessee), and also include the southwest (Texas), midwest (Michigan and Ohio), and mid-Atlantic region (New Jersey). The study protocol, consent forms and questionnaire were approved by the Institutional Review Boards (IRB) at Duke University Medical Center, Baylor College of Medicine, Case Western Reserve University School of Medicine, Louisiana State University, Robert Wood Johnson Medical School/Rutgers Cancer Institute, Wayne State University, the University of Alabama-Birmingham, the Medical University of South Carolina and the University of Tennessee-Knoxville. Additionally, the protocol was approved by central cancer registries in the states of Alabama, Georgia, North Carolina, South Carolina, Tennessee and Texas, SEER (Surveillance, Epidemiology and End Results) registries in New Jersey, Louisiana, and the Detroit metropolitan area, and 9 individual hospital systems in Ohio. Accrual of cases and controls began December 1, 2010 and will be completed by the end of 2015.

Eligible cases include all AA women aged 20 to 79 years newly diagnosed with a histologically confirmed invasive epithelial OVCA since December 1, 2010. Race (full or mixed AA) is based on self-report. Cases are identified through rapid case ascertainment systems that utilize state cancer registries, SEER registries or gynecologic oncology departments at individual hospitals. The physicians of each eligible patient are contacted to request permission to approach the patient. According to the protocol required at each site, either written consent is obtained or consent to contact the women is assumed if the physician does not object within a reasonable period of time (2 to 3 weeks) after notification.

Control identification began in May 2011. An outside contractor (Kreider Research and Consulting) uses list-assisted, random-digit dialing (RDD) to select control women who self-identify as AA race (full or mixed race), and are matched to cases by 5-year age category and state of residence. Phone numbers are chosen from both landline and cellular telephone exchanges. Eligibility is confirmed through a series of screening questions, and contact information for eligible controls is forwarded to the study coordinating center at Duke. Women with a previous diagnosis of OVCA are excluded as are women who have had a bilateral oophorectomy. Only subjects able to complete an interview in English are included.

Cases approved for contact by their physicians and controls identified by the RDD contractor are sent an introductory letter and study brochure with an identifiable study logo. The link to a study website and a toll-free number are also provided to potential study subjects who may have questions about the study. Verbal informed consent is provided by each participant at the time of the telephone interview, and written informed consent is obtained for collection of biospecimens and medical records.

### Data and biospecimen collection

#### Telephone interview

Approximately 1–2 weeks after sending an introductory letter, a trained interviewer contacts the potential study participant by telephone to answer questions and schedule the interview. Women who agree to participate are contacted by telephone at the agreed upon time, and after review of the consent form, a computer-assisted telephone interview (CATI) is administered. The questionnaire includes detailed questions on demographic characteristics, reproductive, gynecologic and medical history, exogenous hormone use (any type of hormone replacement therapy (HRT) and oral contraceptives (OCs)), family history of cancer and lifestyle characteristics such as smoking, alcohol consumption, and physical activity. There are also questions that address constructs that are of particular relevance for this study population including perceived discrimination, cultural and folk beliefs, access to medical care, trust of health care providers and religiosity. A more detailed description of the questionnaire content is provided in Table 1. The CATI surveys for cases are conducted by interviewers at Duke, with the exception of cases from Detroit for which registry policy requires that the interview be completed by a local interviewer. Controls from all sites are interviewed by the interviewers based at Duke and the Karmanos Cancer Institute in Detroit. To increase response rates, an abbreviated, short interview is offered if the study subject expresses a concern about her time spent on the telephone.

**Table 1:** **Data elements in telephone questionnaire, the African American Cancer Epidemiology Study**

Category	Variables
Demographics	Age, education, income, occupation
	Date and place of birth, race, ethnicity, marital status, parents race and place of birth;
Menstrual history	Age at menarche, length and regularity of menstrual cycle; menstrual status, age and reason when (if) periods stopped; menopausal symptoms
Pregnancy history	Number of pregnancies, age and outcome of each pregnancy; breast feeding history for each live birth
Infertility	Difficulty becoming pregnant; Doctor diagnosed infertility and underlying condition; fertility treatment
Contraceptive and hormone use	Birth control method: number of episodes, age of first use and length of use; Ever use of male or female hormones: type of hormone and number of episodes, age at first use and length of use.
Medical and gynecologic history	All variables in the self-reported Charlson Co-Morbidity Index; Hysterectomy; Oophorectomy; Ovarian Cysts; Fibroids; Pelvic Inflammatory Disease; Endometriosis, Polycystic Ovarian Syndrome; Abnormal PAP smear; Ectopic pregnancy; Tubal ligation
Symptoms	Type and length of symptoms including: pelvic abdominal discomfort; change in bowel habits; frequent or painful urination; distended or hard abdomen; lump on abdomen; fatigue or loss of appetite; side or back pains; abnormal bleeding; weight gain, swelling, or water retention; nausea, vomiting, heartburn or indigestion
Medication use	Name of medications for pain or inflammation: underlying condition/ indication, age at first and last use, and length of use of medications
	Name of current prescription medications: underlying condition/indication and length of use
Radiation exposure	X-rays and other imaging procedures: type and age at time of procedure, part of body scanned, and reason for the scan.
Family history of cancer	1st and 2nd degree relatives; age/age at diagnosis
Insurance, access to care	Type of insurance coverage over the last ten years; where medical care was received; access to a regular doctor; interruptions in access to care, receipt of breast and/or cervical cancer screening
Trust in physicians	Trust in physician scale
Social support	Perceived Social Support
Perceived discrimination	Perceived discrimination – major and everyday discrimination
Religiosity and spirituality	Religious services attendance, spirituality, religious affiliation.
Cultural and folk beliefs	Cultural and folk beliefs about fatalism and what causes cancer
Smoking	Smoking status; number of cigarettes per day; age first smoked and number of years smoked; exposure to environmental smoke
Sun exposure	Time spent outdoors by season: summer or spring/winter/fall; Skin effects
Talc use	Regular use of cornstarch, talc, baby or deodorizing powders; age at first use, frequency of use and duration of use, use by sexual partner; occupational exposure to talc or asbestos; lived with someone who worked with talc or asbestos
Height and weight	Self-reported current height and weight; weight gain and loss history; weight at age 18.
Physical activity	Average weekly exercise during the last 12 months (based on International Physical Activity Questionnaire – Short Form); job-related physical activity

#### Food frequency questionnaire

A self-administered food frequency survey (the Block 2005 Food Frequency Questionnaire) is mailed to the study subjects with other study documents. Subjects complete the food frequency questionnaire on their own, but if needed, the interviewer will assist with its completion.

### Biospecimens

In addition to the questionnaires, all study subjects are asked to provide a blood or saliva specimen for DNA analyses. After receiving a signed consent form for specimen collection at the Duke study coordinating center, the information is forwarded to the contractor responsible for specimen collection, Examination Management Service, Inc. (EMSI). EMSI has offices nationwide and arranges for a trained phlebotomist to meet each participant at her home or other convenient location to obtain a biospecimen and anthropometric measurements (height, weight and waist and hip circumferences). Each participant is asked to provide a 30 ml blood sample, however, if she is unable or unwilling to do so, she is asked to give a saliva sample using an Oragene® kit. Oragene® kits are mailed directly to participants who consent to give a biospecimen but do not wish to have a home visit.

Women with OVCA also are asked to grant permission for the study to obtain a formalin-fixed, paraffin-embedded (FFPE) tumor block from their primary tumor. Pathology reports and tumor blocks or sections are requested from pathologists, and the FFPE tumor blocks are cut according to study protocol for all cases. A centralized pathology review for all cases is conducted at Duke by the study pathologist, a specialist in gynecologic cancers.

All study participants are remunerated for their time at two benchmarks during enrollment: 1) upon completion of the telephone interview and 2) upon receipt of either a blood or saliva specimen.

### Follow-up survey

Cases are followed on an annual basis. A follow-up telephone survey is administered by Duke staff and includes questions on insurance, updates to medical history, occupational status, medication use, quality of life, social support, stress and other factors that may be related to outcome. Additionally, medical records are requested to obtain diagnostic, treatment and outcomes information.

### Variables and coding

Demographic characteristics include age at diagnosis for cases and age at interview for controls (categorized as 20- < 40, 40- < 50, 50- < 60, 60- < 70, 70- < 80 years), education (≤high school, some post-high school training, college/graduate degree), annual income (<$10K, $10K- < $25K, $25K- < $50K, $50K- < $75K, ≥$75K), current medical insurance (none, Medicaid, Medicare, other), and access to a private physician (yes, no). Body mass index 1 year before diagnosis (cases)/interview (controls) (BMI) is categorized as <25, 25- < 30, 30- < 35 or ≥35 kg/m^2^. Additional risk factors include parity (0, 1–2, ≥3), months of OC use (never to <3, 3- < 36, 36- < 60, ≥60 months), use of any HRT (ever, never), age at menarche (<12, 12- < 13, ≥13 years), tubal ligation (yes, no), menopausal status (pre-menopausal or postmenopausal), and any first- degree relative with OVCA or breast cancer (yes, no). Premenopausal women are those who are still experiencing menstrual cycles at the date of diagnosis/interview, regardless as to whether the cycles are the usual cycle pattern or missed/interrupted periods. Women who are taking birth control pills are also classified as premenopausal. Women are classified as menopausal if menstrual periods have stopped or both ovaries have been removed. For women < 50 years of age who have had a hysterectomy and do not have menopausal symptoms or have symptoms for less than two years are classified as premenopausal. Women who have had a hysterectomy who are less than 50 years of age and have had symptoms for at least two years or are 50 years of age older are classified as postmenopausal.

Time from diagnosis to ascertainment is calculated as the difference between the date at diagnosis from the pathology report and the date the information is received at the Duke study office. We calculated the number of days from diagnosis to interview as the difference between the date of diagnosis and the date when the telephone interview was completed.

### Statistical analysis

We used descriptive statistics to summarize the characteristics of surveyed AA women. Values are expressed as n (%), means or medians and interquartile ranges. To compare risk factor characteristics between cases and controls we calculated age-adjusted and multivariable-adjusted odds ratios (ORs) and 95 percent confidence intervals (CIs) using unconditional logistic regression analyses. Comparisons of characteristics of responders and non-responders were evaluated with chi-square tests.

Response rates were calculated according to the formula:


Cooperation rates, defined as the proportion of completed interviews among eligible women actually contacted, were calculated according to the formula:


Since this study is ongoing, we calculate the response rate two ways; 1) we assume all pending subjects participate in the study and include the pending subjects in the numerator and 2) we assume all pending subjects decline the study and calculate the rate with the pending subjects in the denominator only.

We collected and managed the subject accrual data using REDCap electronic data capture tools hosted at Duke University [17]. Statistical analyses were performed using SAS version 9.3 (SAS Institute, Cary, NC).

## Results

### Subject accrual

Study enrollment began in May 2011, with cases being deemed eligible if they were diagnosed since December 2010. Our goal is to enroll 850 AA women with invasive epithelial OVCA and 850 controls by December 2015.

As of April 8th, 2014, we identified 1,055 women newly diagnosed with OVCA, of whom 940 met study eligibility criteria. As of this date, 403 newly diagnosed OVCA cases have completed an interview and 68 cases are still pending. Non-participation was due to physician refusals (n = 10), subject refusals (n = 203), death (n = 141) and inability to contact (n = 117). Assuming all pending cases decline to participate in the study, the overall response rate would be 43%. If all pending cases choose to participate the response rate would be as high as 50%. Among cases that we were able to contact, the cooperation rate is 66.5%.

Among 1,334 potential controls identified through RDD, 1,284 met the eligibility criteria. Interviews have been completed with 639 controls and 150 are pending. Non-participation was due to subject refusals (n = 252), inability to contact (n = 240) and death (n = 3). If all pending subjects decline participation in the study, the overall response rate among controls would be 50%, and if all pending subjects agree to participate, the response rate would be 61%. Among potential controls that we were able to contact, the cooperation rate is 72%.

Once participants agreed to be interviewed, the majority completed all components of the study. Among women agreeing to the telephone survey, most (93% of cases and 98% of controls) completed the long questionnaire, which is designed to be completed in approximately 1 hour. A shorter, 15-minute survey, which is offered as an option for women who are unwilling or unable to complete the long questionnaire, was completed by 30 cases and 16 controls.

More than 93% of the women interviewed have also completed the 110-item food frequency questionnaire. Most food frequency questionnaires were self-completed; however, the interviewers completed it on the phone for 15 cases and 24 controls who requested assistance.

To date, 284 (70%) of the enrolled cases and 454 (71%) of the enrolled controls have provided a blood and/or saliva sample. Only 3.7% of the cases and 6.0% of the controls did not consent to biospecimen collection. There are 104 cases and 147 controls who completed the questionnaire and are pending biospecimen collection. Of the samples collected 79% of cases and 78% of controls donated a blood sample.

### Time to ascertainment/interview

The goal of rapid case ascertainment is to identify cancer cases as soon as feasibly possible after diagnosis to minimize loss to death. This is particularly important for diseases like OVCA that have a high fatality rate. Underscoring the severity of OVCA among African-American women, among eligible cases in our study, 15% were deceased at the time of ascertainment.

We examined the time between diagnosis and ascertainment for all identified cases and by participation status. For all OVCA cases, the median time from diagnosis to the receipt of the pathologic information at the study office was 134 days (Table 2). When omitting the first year of accrual to allow for the maturation of the rapid case ascertainment protocols, median days from diagnosis to the identification of cases decreased to 91 days. The median time between diagnosis and ascertainment was longer for non-responders than responders. This difference was especially pronounced for the women who were deceased before they could be contacted. Excluding the first year of accrual, the median days from diagnosis to interview was 145 days or approximately 5 months. More than three-quarters of the OVCA cases are interviewed within 9 months of diagnosis.

**Table 2:** **Time from ovarian cancer diagnosis to ascertainment and interview, the African American Epidemiology Study (AACES), 2010-14**

		Days from diagnosis to ascertainment, all cases		
	N*	Mean	Median	25th-75th percentile range
**Responders**	403	170	126	61-245
**Non-responders**	370	191	159	64-282
Deceased	117	245	226	97-311
MD refusal	9	66	30	26-78
Refusal	163	165	126	60-239
Unable to contact	81	181	140	57-299
**Pending**	62	160	82	48-276
**Total**	835	179	134	62-260
		**Days from diagnosis to ascertainment, omitting cases from first year of ascertainment**		
	N*	**Mean**	**Median**	**25th-75th percentile range**
**Responders**	279	143	91	54-216
**Non-responders**	221	135	93	48-199
Deceased	66	162	132	62-242
MD refusal	5	41	26	20-30
Refusal	101	126	92	45-175
Unable to contact	49	129	73	47-164
**Pending**	59	162	84	51-276
**Total**	559	142	91	50-212
		**Days from diagnosis to interview**		
	N*	**Mean**	**Median**	**25th-75th percentile range**
**All cases**	403	215	178	105-290
**Omitting cases from 1st year of ascertainment**	279	186	145	96-261

*Date of diagnosis was missing for 94 non-responders because some sites that required patient consent before information could be sent to Duke could not send exact diagnosis date for women who did not consent.

### Responder versus non-responder characteristics

Among eligible cases, only a limited number of variables are available to evaluate differences between the 403 responders and 464 non-responders, of which 139 were deceased at ascertainment (Table 3). The responders, on average, were younger than the non-responders, 57 years (standard deviation (SD) = 11.2 years) compared to 61 years (SD = 11.2 years) (p <0.0001). The age at diagnosis for live and deceased non-responders, 60.8 years (SD = 11.2) and 61.2 years (SD = 11.1), respectively, were not statistically different (p = 0.69). Most notably, a smaller proportion of the responders were found in the oldest age categories, 60–69 years and 70–79 years compared to both live and deceased non-responders who appeared to have a similar distribution of age at diagnosis. Stage was more advanced among the non-responders compared to responders, with 84. 2% of deceased non-responders and 61.5% of the live non-responders assigned a stage III/IV at diagnosis compared to 52.2% of the responders. Although the histologic subtype distribution of the responders was similar to that of the live non-responders (p = 0.37), a significant difference in the histologic subtype distribution between responders and deceased non-responders was found. In particular, the serous and endometrioid subtypes were less common among the deceased non-responders compared to both responders and live non-responders. Over 54% of the deceased non-responders were classified as having histology of ‘other’ compared to 15.6% and 21.7% of the responders and live non-responders with the majority of tumors in this category classified as carcinomas NOS (87% overall and 90% of the deceased non-responders, data not shown). The distribution of tumor grade is similar among responders and live non-responders, with just under 75% being poorly-differentiated among both groups. The proportion of poorly-differentiated tumors among the deceased non-responders is higher with approximately 81% classified as poorly-differentiated. However, the distribution of grade was not found to be significantly different from that of the responders (p = 0.20). Although there is only a small proportion of cases with missing histology and stage at diagnosis data, 28% and 55% of live and deceased non-responders versus 17% of responders have missing data for tumor grade. These statistics are preliminary since centralized pathology review is ongoing and grade is missing for a large number of the subjects.

**Table 3:** **Selected characteristics of ovarian cancer cases by responder status, the African American Epidemiology Study (AACES) 2010-14**

Variable	Responders n = 403	Living Non-Responders n = 325	p-value vs. Responders	Deceased Non-Responders n =139	p-value vs. Responders
	**N (%)**	**N (%)**		**N (%)**	
**Age group**					
20-39	26 (6.5)	18 (5.5)		6(4.3)	
40-49	62 (15.4)	37(11.4)		14 (10.1)	
50-59	142 (35.2)	83 (25.5)		29 (20.9)	
60-69	108 (26.8)	106 (32.6)		53 (38.1)	
70-79	65 (16.1)	81 (24.9)	0.0021	37 (26.6)	0.0005
Missing	___	___			
**Histology**					
Clear Cell	8 (2.1)	8 (2.6)		2 (1.5)	
Endometrioid	45 (11.5)	29 (9.4)		0 (0)	
Mucinous	20 (5.1)	17 (5.5)		5 (3.8)	
Serous	242 (62.1)	180 (58.3)		51 (38.9)	
Mixed Cell	14 (3.6)	8 (2.6)		2 (1.5)	
Other	61 (15.6)	67 (21.7)	0.37	71 (54.2)	< 0.001
Missing	13	16		8	
**Stage**					
I/II	182(47.8)	116 (38.5)		19 (15.8)	
III/IV	199 (52.2)	185 (61.5)	0.02	101 (84.2)	< 0.001
Missing	22	24		20	
**Grade**					
Well differentiated	35 (10.5)	31 (13.3)		5 (7.9)	
Moderately differentiated	52 (15.6)	35 (15.0)		5 (7.9)	
Poorly/undifferentiated	246 (73.9)	168 (71.8)	0.61	53 (81.1)	0.20
Missing	70	91		76	

### Descriptive statistics

The mean age at diagnosis (based on the date of the pathology report)/age at interview of the cases and controls, respectively, was 57.4 years (SD = 11.2 years) and 54.1 years (SD = 11.8 years) (p < 0.0001), respectively. Additional comparisons of demographic characteristics and epidemiologic risk factors between cases and controls are found in Table 4. Although the study is designed to frequency match controls to cases by age, there were more cases in the 70–79 year age group than controls, 16.1% versus 8.6% and fewer cases in the youngest age category of 20–39 years compared to controls, 6.5% and 12.4%, respectively. Going forward measures are being taken to focus control identification and recruitment in these older age categories. Response rates for the cases were lower for those 60 years of age and above at diagnosis compared to those below 60 years of age at diagnosis. The age at interview did not appear to be related to response rate among controls (data not shown).

**Table 4:** **Descriptive characteristics of ovarian cancer cases and controls, the African American Cancer Epidemiology Study (AACES), 2010-14†**

Variable	Cases (N = 371)	Controls (N = 622)				
	N (%)	N (%)	OR*	(95% CI)	OR**	(95% CI)
**Age group (years)**						
20-39	23 (6.2)	77 (12.4)				
40-49	61 (16.4)	115 (18.5)				
50-59	131 (35.3)	219 (35.2)				
60-69	97 (26.1)	157 (25.2)				
70-79	59 (15.9)	54 (8.7)				
**Age at menarche (years)**						
<12	83 (22.4)	167 (26.8)	1.00	Referent)	1.00	Referent
12-13	186 (50.1)	300 (48.2)	1.22	(0.88-1.69)	1.30	(0.93-1.80)
13+	102 (27.5)	155 (24.9)	1.27	(0.88-1.84)	1.35	(0.93-1.97)
**Parity**						
0	67 (18.1)	83 (13.3)	1.00	Referent	1.00	Referent
1-2	159 (42.9)	284 (45.7)	0.66	(0.45-0.97)	0.69	(0.47-1.01)
>3	145 (39.1)	255 (41.0)	0.61	(0.41-0.90)	0.61	(0.41-0.91)
**Tubal ligation**						
No	235 (63.5)	375 (60.4)	1.00	Referent	1.00	Referent
Yes	135 (36.5)	246 (39.6)	0.84	(0.64-1.10)	0.96	(0.71-1.28)
Missing	1	1				
**Oral contraceptive use (months)**						
Never to <3	129 (34.8)	145 (23.3)	1.00	Referent	1.00	Referent
3- < 36	102 (27.5)	192 (30.9)	0.64	(0.46-0.91)	0.66	(0.47-0.93)
36 < -60	31 (8.4)	67 (10.8)	0.54	(0.33-0.89)	0.55	(0.33-0.90)
60+	109 (29.4)	218 (35.0)	0.58	(0.42-0.82)	0.58	(0.41-0.82)
**Menopausal status**						
Post-menopausal	266 (72.3)	410 (66.2)	1.00	Referent	1.00	Referent
Pre-menopausal	102 (27.7)	209 (33.8)	1.21	(0.78-1.89)	1.23	(0.78-1.92)
Missing	3	3				
**Hysterectomy**						
No	279 (75.2)	495 (79.6)	1.00	Referent	1.00	Referent
Yes	92 (24.8)	127 (20.4)	1.12	(0.82-1.54)	1.12	(0.82-1.55)
**Use of hormone replacement therapy among women over age > =50**						
No	216 (75.8)	341 (79.7)	1.00	Referent	1.00	Referent
Yes	69 (24.2)	87 (20.3)	1.17	(0.81-1.69)	1.25	(0.86-1.82)
Missing	2	2				
**First degree relative with ovarian cancer**						
No	340 (94.7)	578 (96.8)	1.00	Referent	1.00	Referent
Yes	19 (5.3)	19 (3.2)	1.57	(0.82-3.03)	1.60	(0.82-3.12)
Missing	12	25				
**First degree relative with breast cancer**						
No	281 (77.6)	506 (84.2)	1.00	Referent	1.00	Referent
Yes	81 (22.4)	95 (15.8)	1.46	(1.04-2.04)	1.50	(1.07-2.11)
Missing	9	21				
**First degree relative with ovarian and/or breast cancer**						
No	267 (73.8)	491 (81.7)	1.00	Referent	1.00	Referent
Yes	95 (26.2)	110 (18.3)	1.50	(1.09-2.06)	1.53	(1.11-2.11)
Missing	9	21				
**Body mass index 1 year before diagnosis (kg/m** ^**2**^ **)**						
<24.9	54 (14.6)	119 (19.2)	1.00	Referent	1.00	Referent
25–29.9	95 (25.7)	157 (25.3)	1.24	(0.82-1.88)	1.31	(0.86-1.99)
30–34.9	107 (29.0)	157 (25.3)	1.49	(0.99-2.24)	1.50	(0.99-2.27)
> = 35	113 (30.6)	188 (30.3)	1.29	(0.86-1.92)	1.27	(0.85-1.91)
Missing	2	1				
**Education**						
High school or less	173 (46.6)	223 (35.9)	1.00	Referent	1.00	Referent
Some post-high school training	115 (31.0)	227 (36.6)	0.69	(0.51-0.93)	0.69	(0.51-0.95)
College or graduate degree	83 (22.4)	171 (27.5)	0.66	(0.48-0.93)	0.66	(0.46-0.95)
Missing		1				
**Annual Income**						
< $10,000	85 (23.4)	130 (21.2)	1.00	Referent	1.00	Referent
$10,000 - < $25,000	98 (26.9)	145 (23.6)	1.00	(0.68-1.46)	1.04	(0.71-1.53)
$25,000 - < $50,000	88 (24.2)	131 (21.3)	1.04	(0.71-1.54)	1.08	(0.72-1.60)
$50,000 - < $75.000	48 (13.2)	103 (16.8)	0.72	(0.46-1.12)	0.75	(0.48-1.19)
≥$75,000	45 (12.4)	105 (17.1)	0.66	(0.42-1.04)	0.73	(0.46-1.17)
Missing	7	8				
**Private Physician**						
No	104 (29.1)	158 (26.2)	1.00	Referent	1.00	Referent
Yes	253 (70.9)	445 (73.8)	0.77	(0.57-1.03)	0.77	(0.57-1.05)
Missing	14	19				
**Current Insurance**						
None	41 (11.1)	76 (12.3)	1.00	Referent	1.00	Referent
Medicaid	97 (26.1)	124 (20.0)	1.37	(0.86-2.20)	1.38	(0.85-2.22)
Medicare	96 (25.9)	128 (20.6)	1.04	(0.63-1.74)	1.03	(0.61-1.72)
Other Insurance	137 (36.9)	292 (47.1)	0.84	(0.54-1.29)	0.86	(0.56-1.34)
Missing	0	2				
**Ever smoked**						
Current/Former	163 (43.9)	259 (41.6)	1.00	Referent	1.00	Referent
Never	208 (56.1)	363 (58.4)	0.97	(0.75-1.27)	1.00	(0.76-1.30)

†Forty-nine patients (32 cases and 17 controls) were excluded from this table due to missing data for either parity or months of oral contraceptive use.

OR = Odds Ratio, CI = Confidence Interval.

*Age adjusted(ORs).

**Multivariable adjusted (ORs) adjusted for age, months of oral contraceptive use, and parity.

Age-adjusted and multivariable adjusted analyses of well-established OVCA risk factors revealed associations that were in the expected direction, although not all were statistically significant (Table 4). Few differences in age-adjusted ORs compared to multivariable-adjusted ORs are seen. As compared to controls, cases were less likely to have used OCs with a weak inverse trend in reduced risk with longer duration of use. Compared to controls cases also were less likely to have had a tubal ligation, but were more likely to be nulliparous, have a relative with breast or ovarian cancer, or have used any type of HRT. Case–control associations with BMI 1 year prior to the referent date of 30- < 35 kg/m^2^, parity > 3, months of OC use, and family history of breast or ovarian cancer approached or were statistically significant.

In Table 4, preliminary case–control comparisons of variables related to socio-economic status revealed that controls were more likely to report having some post high school training or a college education compared to cases. Although not reaching statistical significance, controls were more likely to have an annual income of $75,000 or more and more controls reported having access to a private physician compared to cases. No difference was found with the current insurance in cases compared to controls although there was a tendency for cases to be more likely to report they had ‘Medicaid’ and less likely to report ‘Other Insurance’ versus ‘No Insurance’ compared to controls.

In Table 5, we present both age-adjusted and multivariable adjusted ORs and 95% CIs for case–control associations with BMI, education and income for advanced (III/IV) versus early (I/II) stage at diagnosis. Few differences in the age-adjusted and multivariable-adjusted ORs are seen, with the exception of annual income, where the multivariable-adjusted ORs show an inverse association with early stage ovarian cancer cases but not advanced stage cases compared to controls. A case-only analysis using multivariable logistic regression, adjusting for age at diagnosis, does not support that indicators of socio-economic status along with BMI are associated with advanced stage at diagnosis, an important prognostic indicator (data not shown).

**Table 5:** **Odds ratios (OR) and 95% confidence (CIs) intervals for case–control associations with BMI, education and annual income for early and advanced stage ovarian cancer, the African American Cancer Epidemiology Study (AACES), 2010-14†**

	Early Stage at Diagnosis (Stage I/II)						Advanced Stage at Diagnosis (Stage III/IV)				
Variable	Controls (N = 622)	Cases (N = 168)					Cases (N = 182)				
	N (%)	N (%)	OR*	(95% CI)	OR**	(95% CI)	N (%)	OR*	(95% CI)	OR**	(95% CI)
**Body mass index 1 year before diagnosis (kg/m** ^**2**^ **)**											
<24.9	119 (19.2)	25 (14.9)	1.00	Referent	1.00	Referent	26 (14.4)	1.00	Referent	1.00	Referent
25–29.9	157 (25.3)	38 (22.6)	1.10	(0.63-1.93)	1.18	(0.66-2.08)	50 (27.6)	1.33	(0.77-2.27)	1.31	(0.76-2.26)
30–34.9	157 (25.3)	51 (30.4)	1.53	(0.90-2.62)	1.56	(0.90-2.68)	51 (28.2)	1.46	(0.85-2.49)	1.45	(0.84-2.48)
> = 35	188 (30.3)	54 (32.1)	1.33	(0.78-2.26)	1.33	(0.78-2.28)	54 (29.8)	1.31	(0.77-2.22)	1.23	(0.72-2.09)
Missing	1						1				
**Education**											
High school or less	223 (35.9)	80 (47.6)	1.00	Referent	1.00	Referent	84 (46.2)	1.00	Referent	1.00	Referent
Some post-high school training	227 (36.6)	47 (28.0)	0.60	(0.40-0.89)	0.59	(0.39-0.90)	62 (34.1)	0.78	(0.53-1.14)	0.84	(0.56-1.25)
College or graduate degree	171 (27.5)	41 (24.4)	0.69	(0.45-1.06)	0.65	(0.41-1.04)	36 (19.8)	0.59	(0.38-0.93)	0.67	(0.42-1.09)
Missing	1										
**Annual Income**											
< $10,000	130 (21.2)	43 (25.9)	1.00	Referent	1.00	Referent	37 (20.8)	1.00	Referent	1.00	Referent
$10,000 - < $25,000	145 (23.6)	41 (24.7)	0.85	(0.52-1.39)	0.86	(0.52-1.43)	49 (27.5)	1.15	(0.70-1.89)	1.19	(0.72-1.96)
$25,000 - < $50,000	131 (21.3)	41 (24.7)	0.96	(0.59-1.58)	0.96	(0.58-1.60)	43 (24.2)	1.17	(0.70-1.94)	1.27	(0.75-2.13)
$50,000 - < $75.000	103 (16.8)	23 (13.9)	0.68	(0.38-1.20)	0.69	(0.38-1.25)	23 (12.9)	0.79	(0.44-1.42)	0.91	(0.49-1.67)
≥ $75,000	105 (17.1)	18 (10.8)	0.52	(0.28-0.96)	0.54	(0.29-1.02)	26 (14.6)	0.90	(0.51-1.59)	1.09	(0.60-1.99)
Missing	8	2					4				

†Seventeen controls were excluded from this table; 14 cases excluded from the early stage cases and 17 cases excluded from advanced stage cases due to missing data for either parity or months of oral contraceptive use.

*Age adjusted(ORs).

**Multivariable adjusted (ORs) adjusted for age, months of oral contraceptive use, and parity.

## Discussion

The progress to date on the AACES study demonstrates both the importance and the challenge of studying OVCA in AA women. The high proportion of women who are deceased before they could be enrolled in the study underscores the severity of the disease in this population and the urgent need to better understand factors that affect its etiology and prognosis. The high frequency of rapidly fatal disease also highlights one of the challenges of conducting an epidemiologic study of OVCA in this population.

The possible selection bias that can result from low participation rates in case–control studies is a topic of high concern and has been discussed repeatedly in the literature [18, 19]. In addition to secular trends of declining participation rates across all types of studies, [18] AACES faces the additional challenges of the typically lower participation rates among minority populations and lower participation rates among cases due to advanced disease.

Although, many case–control studies report higher response rates among cases than controls [13, 19], the opposite is true in AACES, which is likely attributable to disease severity. As our data show, non-responders, particularly those patients who are deceased at ascertainment, are more likely to be diagnosed with advanced stage disease. Although no differences in tumor histology or grade were observed in responders compared to live-non-responders, differences were found in the deceased non-responders. These are likely due to a higher proportion of tumors defined as carcinoma NOS and may explain why there are fewer serous and endometrioid cases among this group of women, who may have not received the same level of care or pathologic scrutiny.

Despite working with institutions that have established methods for rapid case ascertainment, including three SEER sites, we have a remarkably high proportion of cases deceased at the time of accrual (15% of the total eligible). The proportion of deceased cases in AACES is approximately the same as what we observed among AA women with invasive OVCA in a previous population-based study of OVCA in North Carolina (14%) [13] (unpublished data). Notably, the overall proportion of deceased cases in that study was 4%, but was approximately three times higher in AAs than whites. In AACES, we found a longer length of time between date of diagnosis and ascertainment among those deceased as compared to those who eventually enrolled in the study, which may partially account for the overall high proportion of deceased patients. In addition, we suspect the relatively high proportion of cases that we were unable to contact (12% of eligible cases) also may be related to disease severity as we have heard anecdotal accounts that some women have had to move in with someone else to receive care after their diagnosis. Although it is possible that the high prevalence of BMI and low socioeconomic status may contribute to the high proportion of deaths at ascertainment, we were not able to detect an association between these factors and advanced stage at diagnosis, an important prognostic indicator. BMI and factors related to socio-economic status were not available for the non-responders, limiting our ability to assess how these factors may have influenced the proportion of deaths among eligible cases prior to ascertainment.

Our ability to evaluate selection bias among the OVCA cases was limited, with data available for only a few patient characteristics for the non-responders. Overall, the non-responders are older and appear to have more advanced disease, and likely a poorer prognosis, compared to responders. This finding is found among similarly designed studies as AACES, although not consistently [19].

Because the target population is AA women with OVCA, a multi-state, multi-center, case–control study was the only viable study design that would permit the accrual of sufficient numbers of cases and controls in a reasonable period of time. In spite of the limitations and challenges of AACES, epidemiologic risk factors appear to be distributed as would be expected from previous published reports, with no history of OC use, nulliparity, no history of tubal ligation and a family history of breast or OVCA more common among cases than controls [13, 20, 21]. Although more refined analyses are required in a larger sample, a noteworthy finding is the increased risk associated with a BMI greater than 30 kg/m^2^ with ORs in the range of 1.4 to 1.5. The high prevalence of obese and severely obese women in this population (~55% of the control women) suggests reducing BMI may be an effective means of preventing OVCA among AAs.

Upon completion, AACES will represent the largest study to date of OVCA in AA women, with more than five times as many cases as in any previous study. The number of AA cases that we will enroll will exceed even the number of cases from large consortia such as the Ovarian Cancer Association Consortium (382 invasive cases in women of African ancestry across more than 60 individual studies in the U.S. and Europe) or the African American Cohort Consortium (~240 cases in 5 cohorts) (personal communication J. Palmer). The major strengths of the AACES study are that it uses standard protocols for data collection across 10 geographic regions in the U.S. encompassing both rural and urban regions and it collects data that are of particular relevance for AA women, including perceived discrimination, access to health care, and cultural and folk beliefs, that have not been collected in previous studies of OVCA.

Although the main limitation of the AACES appears to lie in the possible but unavoidable selection bias, the large study sample and the information collected represent a rich opportunity for studying an uncommon cancer in a minority population. The AACES telephone surveys and food frequency questionnaires, the biospecimens and the medical record data will provide an unprecedented resource, both in breadth and depth, for studying OVCA in women of African ancestry in the U.S.

AACES participants represent an understudied population, with a disproportionate number of women of lower socio-economic status. It is well documented that women of African descent in the U.S. experience significant health disparities leading to poorer outcomes for many diseases. We are confident that AACES will lead to an increased understanding of the factors that influence risk and overall outcome of this disease among AA women.

## Conclusion

Most of the epidemiologic risk factor associations in AA women were found to be similar to those reported for white women. Our data support that obesity, found to be prevalent in more than 60% of the cases, was significantly associated with increased OVCA risk. This finding suggests the potential for an effective intervention on the risk in AAs. A high proportion of women with OVCA was deceased and among these women, a high proportion was diagnosed at an advanced stage. Since early stage cancers have a better survival, there is a clear need to better understand causes of advanced stage cancer diagnoses and to address access to care issues in this population. Upon completion of enrollment, AACES will be the largest epidemiologic study of OVCA in AA women, providing a unique opportunity to increase our knowledge of the epidemiology of OVCA in AA women.

## Abbreviations

AA: African American
AACES: African American Cancer Epidemiology Study
BMI: Body mass index
CI: Confidence interval
CATI: Computer assisted telephone interview
HRT: Hormone replacement therapy
IRB: Institutional Review Board
OR: Odds ratio
OCs: Oral contraceptives
OVCA: Ovarian cancer
RDD: Random-digit dialing
SD: Standard deviation
SEER: Surveillance, Epidemiology and End Results.
